# Supplementary material for: Resveratrol Ameliorates Chronic Stress in Kennel Dogs and Mice by Regulating Gut Microbiome and Metabolome Related to Tryptophan Metabolism
Source: Antioxidants (Basel). 2025 Feb 9;14(2):195. doi: 10.3390/antiox14020195 (PMC11851397; doi:10.3390/antiox14020195)
Supplement: Supplementary file 1 [file antioxidants-14-00195-s001.zip › antioxidants-3435680-supplementary.pdf]

antioxidants

MDPI

Table S1. The ingredients and analyzed chemical composition of the basal diet for the dogs.

Items	**Basal Diet**
Ingredients (as-is basis)	
Chicken meal	32.00%
Duck meal	20.00%
Sweet potato meal	12.00%
Tapioca meal	12.00%
Brewer's yeast meal	5.00%
Alfalfa grass granules	5.00%
Chicken oil	3.00%
Fish oil	2.00%
Flaxseed	1.00%
Pumpkin meal	1.00%
Apple meal	0.50%
Analyzed composition	
DM (%)	96.31%
CP (% DM)	37.27%
EE (% DM)	18.12%
CF (% DM)	2.61%

Premix: lysine, DL-methionine, fructooligosaccharides, vitamin A, vitamin D3, vitamin E, vitamin B1, vitamin B2, vitamin B6, vitamin B12, D-biotin, D-pantothenic acid, folic acid, niacin, ferrous sulfate, copper sulfate, manganese sulfate, zinc sulfate, calcium iodate, sodium selenite, and calcium hydrogen phosphate. DM, dry matter; CP, crude protein; EE, ether extract; CF, crude fiber.

Table S2. Dog behaviors assessed in OFT.

Behavioral Parameter	Definition	Measurement Duration (s) Frequency (time)
Immobility	Dog keeps its body still (e.g., standing, sitting, or lying).	Duration
Escape attempt	Dog's body approaches the exit point with eyes focused on it.	Duration
Exploratory behavior	Sniffing objects in the surrounding and touching objects with nose, mouth, or paws.	Duration
Vocalization	Rough sound repeats in quick succession, deep threatening rumble, or sustained whimper.	Frequency
Pace	Repeating walk or running in circles without clear purpose.	Duration

Table S3. Primer sequences.

Gene	GenBank® Accession Number	Forward Sequence	Reverse Sequence
GAPDH	NM_001289726.2	CCTCGTCCCGTAGACAAAATG	TGAGGTCAATGAAGGGGTCGT
Claudin-1	NM_016674.4	AGTAACTTTGCCATATAAGACCA	AAAGTGCAAAGACCGTC
CYP1A1	NM_001136059.2	CCTCATGTACCTGGTAACCA	AAGGATGAATGCCGGAAGGT

CYP1B1	NM_001364889.1	GGATATCAGCCACGAC-GAAT	ATTATCTGGGCAAAGCAACG
SLC7A8	NM_016972.2	GGTCTCCCAACTTCCTAC-CTAGCT	GAGAGAGAGAGAGACAA-TAATTCAAGGAG
SLC16A10	NM_001114332.1	CGCCTACGGGGTGCTCTTC	ACTCACGATGGGGCAGCAG
SLC6A19	NM_001359603.1	GCCACTGAGCGCTTTGATG	GCCTCAAAGTTCTCTGAAGTCACA

Table S4. Standard compounds of metabolites in tryptophan metabolism pathway.

Abbreviation	Compound Name	HMDB
3-HAA	3-Hydroxyanthranilic acid	HMDB0001476
5-HIAA	5-Hydroxyindoleacetic acid	HMDB0000763
5-HT	5-Hydroxytryptamine	HMDB0000259
5-HTP	5-Hydroxytryptophan	HMDB0000472
IA	Indole acrylic acid	HMDB0000734
IAA	Indole-3-acetic acid	HMDB0000197
IAAld	Indole-3-acetaldehyde	HMDB0001190
IAld	Indole-3-aldehyde	HMDB0029737
IAM	Indole-3-acetamide	HMDB0029739
IE	Indole ethanol	HMDB0003447
ILA	Indole-3-lactic acid	HMDB0000671
Indole	Indole	HMDB0000738
IPA	3-Indolepropionic acid	HMDB0002302
IPYA	Indole-3-pyruvate	HMDB0060067
KNA	Kynurenic acid	HMDB0000715
Kyn	Kynurenine	HMDB0000684
MLT	Melatonin	HMDB0001389
NAS	N-Acetyl-5-hydroxytryptamine	HMDB0001238
PA	Picolinic acid	HMDB0002243
QA	Quinolinic acid	HMDB0000232
Skatole	Skatole	HMDB0000466
TAM	Tryptamine	HMDB0000303
Trp	Tryptophan	HMDB0000929

Table S5. Differential metabolites between Con and Res groups in serum at T2 in dogs.

KEGG	Name	Tendency (Res/Con)
C10245	Astringin	Up
C10166	Piplartine	Up

C01752	Scopoletin	Up
NA	Cefditoren	Up
C09314	Trioxsalen	Up
C03771	2-Oxoarginine	Up
C01035	4-Guanidinobutanoic acid	Up
C01297	6-Hydroxypseudooxynicotine	Up
NA	Neotame	Up
C08491	Jasmonic acid	Up
C13044	Tricaprilin	Up
C08278	Suberic acid	Up
C08449	Falcarindiol	Up
NA	Chloropyramine	Up
C00009	Phosphoric acid	Up
C08021	Remifentanil	Up
NA	Cholic acid glucuronide	Up
C01717	Kynurenic acid	Up
C14214	Dibutyl phthalate	Up
C17240	p-Hydroxybenzyldesulphoglucosinolate	Up
C09695	Lacinilene C 7-methyl ether	Up
C02105	(S)-Reticuline	Up
C00530	Hydroquinone	Up
NA	Diethyl malonate	Up
NA	Alanyl-Tyrosine	Down
NA	Glycerol 1-octadecanoate	Down
NA	Beta-D-Glucopyranuronic acid	Down
C07890	Midodrine	Down
NA	Acetyl tributyl citrate	Down
C03972	Tetrahydrodipicolinate	Down
NA	Indecainide	Down
C14216	Bisphenol S	Down
C14396	Isopropylbenzene	Down
NA	Butalbital	Down
C07428	Phenacemide	Down
NA	Polyoxyethylene 40 monostearate	Down
C13828	Dihomo-gamma-Linolenoyl ethanolamide	Down
C04911	Ganglioside GM1 (18:1/12:0)	Down
C16300	Stearidonic acid	Down
C11301	Sulfolithocholylglycine	Down
C08313	3-Methylindole	Down
NA	Linolenelaidic acid	Down
NA	Valyl-Serine	Down
C13008	Demethylated antipyrine	Down
NA	3-Hydroxyhexadecadienoylcarnitine	Down
C08896	Digitogenin	Down
C02564	N-Feruloylglycine	Down
C04554	3a,7a-Dihydroxycoprostanic acid	Down
C15793	2-Deoxycastasterone	Down
NA	Indoxyl sulfate	Down
C07624	Abacavir	Down
C08073	Natamycin	Down
C10462	Gingerol	Down

NA	N1-(2,4-Dimethoxybenzyl)-n2-(2-(pyridin-2-yl) ethyl)oxalamide	Down
C07523	Propofol	Down
C14762	13S-hydroxyoctadecadienoic acid	Down
NA	Arachidonoyl Serinol	Down
C04884	Ganglioside GM2 (d18:1/12:0)	Down
NA	3, 5-Tetradecadiencarnitine	Down
NA	Homovanillic acid sulfate	Down
C08858	convallatoxin	Down
NA	9,12-Hexadecadienoylcarnitine	Down
NA	N-Lauroylglycine	Down
C09264	Aesculin	Down
NA	cis-5-Tetradecenoylcarnitine	Down
C01192	1-palmitoylglycerone 3-phosphate	Down
C00350	PE(16:0/16:0)	Down
C13828	PGF2a ethanolamide	Down
C17419	Soyasapogenol A	Down
C06475	Prostaglandin F1a	Down
C03033	3-Methoxy-4-hydroxyphenylglycol glucuronide	Down
C02165	Leukotriene B4	Down
C06428	Eicosapentaenoic acid	Down
C06574	Ampicillin	Down
C02394	Cinnamyl alcohol	Down
NA	Propylparaben	Down
C00010	Coenzyme A	Down
C10438	Cinnamic acid	Down
C04230	LysoPC(20:5(5Z,8Z,11Z,14Z,17Z))	Down
C14291	Carbofuran	Down
C07113	Acetophenone	Down

Table S6. Differential metabolites between Con and Res groups in feces at T2 in dogs.

KEGG	Name	Tendency (Res vs. Con)
C03582	Resveratrol	Up
C03882	Piperine	Up
NA	Fenoterol	Up
NA	Acenocoumarol	Up
C06317	4-Hydroxy-3-methoxybenzenemethanol	Up
C06991	Etodolac	Up
C10166	Piplartine	Up
C02916	(R,S)-Norlaudanosoline	Up
C18295	Pretetramid	Up
C08163	Letrozole	Up
C11512	Methyl jasmonate	Up
NA	Histidinyl-Phenylalanine	Up
C02046	Atropine	Up
C01517	Naproxen	Up
NA	Asparaginyl-Arginine	Up
C06628	4-Hydroxy-6-methylpretetramide	Up
C06171	Codeinone	Up
C00472	Quinone	Up

C01699	Gibberellin A3	Up
C16199	5-Sulfosalicylic acid	Up
NA	Guaifenesin	Up
C01617	Taxifolin	Up
C06563	Genistein	Up
NA	Artemether	Up
C00530	Hydroquinone	Up
C07042	Hydromorphone	Up
C05631	Eriodictyol	Up
C01405	Aspirin	Up
C10193	Tricin	Up
C09311	Scoparone	Up
C00539	Benzoic acid	Up
C00774	Phloretin	Up
C05841	Nicotinate D-ribonucleoside	Up
C07235	Rifabutin	Up
C01424	Gallic acid	Up
NA	Succinyladenosine	Up
C00788	Epinephrine	Up
C15584	Phenol	Up
NA	3-(3,4-Dimethoxyphenyl)-2-propenoic acid	Up
C01134	Pantetheine 4'-phosphate	Up
C15572	Guaiacol	Up
C05144	(beta-D-Mannuronate)n	Up
C07319	Sumatriptan	Up
C09842	Chrysanthemic acid	Up
C08271	L-Coprine	Up
C00196	2-Pyrocatechuic acid	Up
C07461	Chlorothiazide	Up
C07710	Telmisartan	Up
C05598	Phenylacetylglycine	Up
C06825	Amlodipine	Up
NA	Glipizide	Up
NA	3-Nitrotyrosine	Up
NA	Valyl-Methionine	Up
C07056	Isoproterenol	Up
NA	APGPR Enterostatin	Up
C00078	L-Tryptophan	Up
C01657	N-Acetyl-L-tyrosine	Up
C02946	4-Acetamidobutanoic acid	Up
NA	N6-Carbamoyl-L-threonyladenosine	Up
C06174	Codeine	Up
C07142	Tocainide	Up
NA	Eletriptan	Up
C00755	Vanillin	Up
C01108	1,2,3-Trihydroxybenzene	Up
C07010	Fluphenazine	Up
C01746	Piperidine	Up
C07080	Loperamide	Up
NA	Cortolone-3-glucuronide	Up
NA	Arginyl-Phenylalanine	Up

C18555	Benzofenap	Up
C00152	L-Asparagine	Up
C05588	Metanephrine	Up
NA	Vorinostat	Up
C01772	2-Hydroxycinnamic acid	Up
NA	Alosetron	Up
C02505	2-Phenylacetamide	Up
C06199	Hordenine	Up
C05565	Hydantoin-5-propionic acid	Up
NA	Suspensolide F	Up
NA	Methionyl-Leucine	Up
C00064	L-Glutamine	Up
C00955	Tryptophanol	Up
C01161	3,4-Dihydroxybenzeneacetic acid	Up
C07220	Mexiletine	Up
C04858	Apiin	Up
C06924	Clozapine	Up
C10447	3,4-Dihydroxyhydrocinnamic acid	Up
C00082	L-Tyrosine	Up
NA	Hirsutin	Up
C14512	Benzofuran	Up
C03519	N-Acetyl-L-phenylalanine	Up
C09993	Kanokoside A	Up
C06536	Harmaline	Up
C10438	trans-Cinnamic acid	Up
C05332	Phenylethylamine	Up
C00079	L-Phenylalanine	Up
C17821	gamma-Asarone	Up
C00858	Formononetin	Up
NA	Lysyl-Phenylalanine	Up
C08336	Neolinustatin	Up
NA	2-Hexenoylcarnitine	Up
C17846	trans-Isoasarone	Up
C00418	Mevalonic acid	Up
C02961	Cytarabine	Up
C12650	Capecitabine	Up
C00431	5-Aminopentanoic acid	Up
NA	Valyl-Valine	Up
C17231	3-(7'-Methylthio)heptylmalic acid	Up
C07819	Dyphylline	Up
C08964	Calenduloside E	Up
NA	Acetylglycine	Up
NA	Troxerutin	Up
NA	Tyrosyl-Tyrosine	Up
C18260	1,6-Dimethoxypyrene	Up
C02191	Protoporphyrin IX	Up
C11332	Leucyl-Leucine	Up
C19506	Styrene	Up
C00385	Xanthine	Down
NA	Lewis X trisaccharide	Down
C02427	Homocitrulline	Down

C07921	Metyrosine	Down
C03145	N-Formyl-L-methionine	Down
C06949	Diazoxide	Down
C02714	N-Acetylputrescine	Down
NA	Mesalazine	Down
C06873	Carmustine	Down
C00584	Prostaglandin E2	Down
NA	Alanyl-Valine	Down
C02214	Glutaconic acid	Down
NA	Alprenolol	Down
C06804	Acetaminophen	Down
C08896	Digitogenin	Down
C06608	Diethyl phosphate	Down
C12144	Phytosphingosine	Down
C00009	Phosphoric acid	Down
NA	Retinyl beta-glucuronide	Down
NA	Diethyl tartrate	Down
NA	Acepromazine	Down
NA	9,12-Hexadecadienoylcarnitine	Down
NA	2-Methyl-1,4-naphthalenediol bis (dihydrogen phosphate)	Down
NA	Cernuine	Down
C00262	Hypoxanthine	Down
C05487	17alpha,21-Dihydroxypregnenolone	Down
C07608	Hyperforin	Down
NA	Anacardic acid	Down
C01692	Disulfiram	Down
C00735	Cortisol	Down
C01478	Arsenate	Down
C02174	PPPi	Down
C00571	Cyclohexylamine	Down
C17369	Northienamycin	Down
C08159	Anastrozole	Down
C09071	Cascarillin	Down
C08773	Nomilin	Down
C16605	2H-Dibenz[b,f]azepin-2-one	Down
NA	Lysyl-Valine	Down
C09826	Pinobanksin	Down
C12580	Trilostane	Down
C17854	(S)-3-Butyl-1(3H)-isobenzofuranone	Down
D02527	Aminopterin	Down
C07826	Aprobarbital	Down
C11994	10-Deoxymethymycin	Down
C18620	3-Hydroxypicolinic acid	Down
C09282	(R)-Oxypeucedanin	Down
C01120	Sphinganine 1-phosphate	Down
C01835	Maltotriose	Down
C11758	Mupirocin	Down
C11208	Iprodione	Down
C00355	L-Dopa	Down
C06593	epsilon-Caprolactam	Down

C16362	3,6,8-Trimethylallantoin	Down
C02997	N-Acetylhistidine	Down
C00841	Xanthotoxol	Down
C11457	3-(3-Hydroxyphenyl) propanoic acid	Down
C09622	Botrydial	Down
C00330	Deoxyguanosine	Down
C10462	Gingerol	Down
C06861	Buspirone	Down
NA	Amidosulfonic acid	Down
C07643	4-Hydroxycyclophosphamide	Down
C00624	N-Acetylglutamic acid	Down
C00163	Propionic acid	Down
C18165	Enterolactone	Down
NA	2-(4-Allyl-2,6-dimethoxyphenoxy)-1-(3,4,5-trimethoxyphenyl)-1-propanol	Down
C14860	2,2-Dichloro-1,1-ethanediol	Down
C00059	Sulfate	Down
NA	Tenofovir	Down
NA	Varanic acid	Down
C02633	Crustecdysone	Down
NA	6-Amino-9H-purine-9-propanoic acid	Down
C08793	Cucurbitacin A	Down
NA	Halobetasol Propionate	Down
C08876	neriifolin	Down
C07415	Paroxetine	Down
C00815	Citramalic acid	Down
NA	Allopurinol	Down
C14149	N-Cyclopropylammelide	Down
C05465	Taurochenodesoxycholic acid	Down
C08798	Cucurbitacin F	Down
NA	3b-Hydroxy-5-cholenoic acid	Down
NA	Acetyl tributyl citrate	Down
C00526	Deoxyuridine	Down
C06427	Alpha-Linolenic acid	Down
C03033	Pregnanediol-3-glucuronide	Down
NA	Ganciclovir	Down
C01029	N8-Acetylspermidine	Down
C05643	6-Hydroxymelatonin	Down
C00106	Uracil	Down
C00181	D-Xylose	Down
C00308	Canavanine	Down
NA	Posaconazole	Down
C01737	Neomycin	Down
C05512	Deoxyinosine	Down
NA	Arbekacin	Down
NA	Fesoterodine	Down
C07215	2-Methylbenzoic acid	Down
C06543	Solanidine	Down
NA	2,6-Dimethoxybenzoic acid	Down
C17226	2-(6'-Methylthio) hexylmalic acid	Down
C15643	Dexamethasone	Down

NA	Bortezomib	Down
C12811	Allylestrenol	Down
NA	Secobarbital	Down
C09980	Yangonin	Down
C07309	Sotalol	Down
C00257	Gluconic acid	Down
C19670	Oleamide	Down
C10544	8-Acetoxypinoresinol	Down
C00319	Sphingosine	Down
C11277	Adefovir Dipivoxil	Down
C07521	Thiopental	Down
C01132	N-Acetyl-D-galactosamine	Down
C04643	7-Ketodeoxycholic acid	Down
C13828	Dihomo-gamma-Linolenoyl ethanolamide	Down
C00388	Histamine	Down
C00315	Spermidine	Down
NA	Methyl bisnorbiotinyl ketone	Down
NA	8-Hydroxy-deoxyguanosine	Down
C03410	N-Glycolylneuraminic acid	Down
C08898	Diosgenin	Down
C00463	Indole	Down
C17217	Dihomomethionine	Down
NA	Resolvin D1	Down
NA	Calcitroic acid	Down
NA	Moclobemide	Down
C00645	N-Acetylmannosamine	Down
NA	6-O-Desmethyldonepezil	Down
C01921	Glycocholic acid	Down
C18166	Enterodiol	Down
C08340	Sarmentosin	Down
C02713	N-Acetylmuramic acid	Down
C14441	16-Oxoestrone	Down
C02154	Glyceraldehyde	Down
NA	Alpha-Hydroxyisobutyric acid	Down
C00352	Glucosamine 6-phosphate	Down
C17277	Valencene	Down
NA	Amcinonide	Down
C16759	S-Allylcysteine	Down
C04218	alpha,alpha'-Trehalose 6-mycolate	Down
C02775	2,3-Dihydroxyindole	Down
C01909	Dethiobiotin	Down
C18226	ent-Cassa-12,15-diene	Down
NA	Leukotriene E3	Down
NA	S-Nitrosoglutathione	Down
C19525	Sudan I	Down
C00762	Cortisone	Down
C01451	Salicin	Down
NA	Levulinic acid	Down
NA	Roxatidine acetate	Down
C03045	2-Hydroxystearic acid	Down
NA	Methylprednisolone	Down

NA	para-Trifluoromethylphenol	Down
C05793	L-Urobilin	Down
C05629	Hydrocinnamic acid	Down

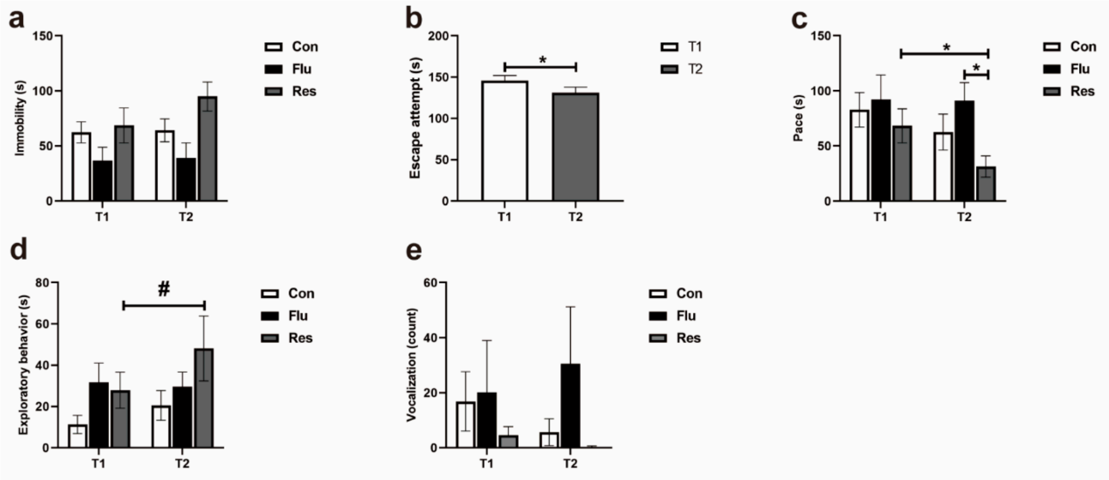

Figure S1. Effects of treatment administrations on behaviors in the OFT in the dogs: (**a**) immobility; (**b**) escape attempt; (**c**) pace; (**d**) exploratory behavior; (**e**) vocalization. Data are presented as the mean ± SEM. The symbol (*) indicates a statistically significant difference between two groups (*$P < 0.05$), and the symbol (#) represents a difference tendency ($P < 0.10$).

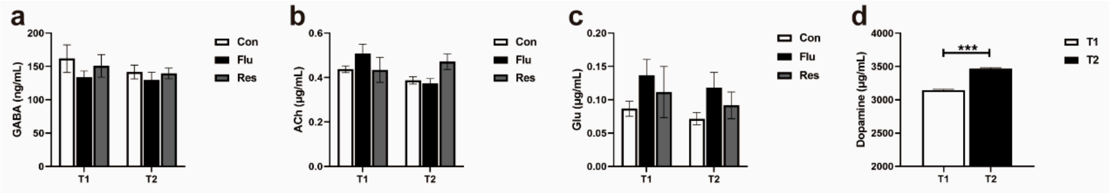

Figure S2. Effects of treatment administrations on neurotransmitters at T1 and T2 in the dogs: (**a**) GABA, γ-aminobutyric acid; (**b**) ACh, acetylcholine; (**c**) Glu, glutamate; (**d**) dopamine. Data are presented as the mean ± SEM. The symbol (*) indicates a statistically significant difference between two groups (*** $P < 0.001$).

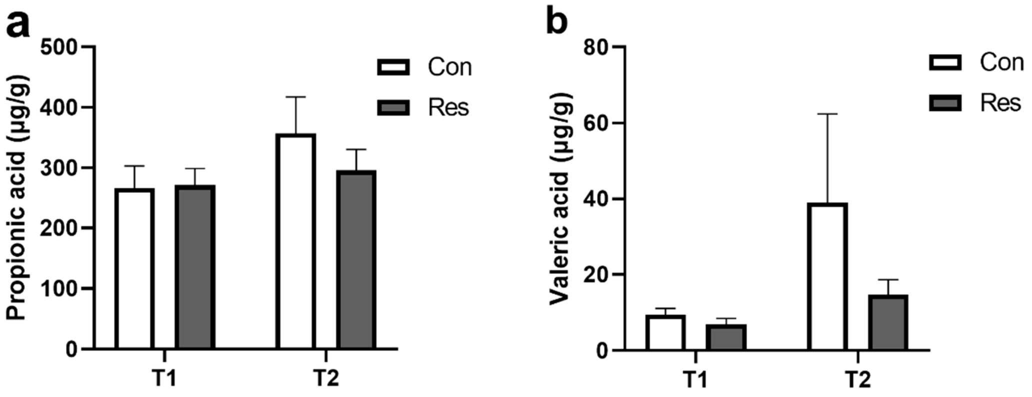

Figure S3. Effects of resveratrol on fecal SCFAs and BCFAs in the dogs: (**a**) propionic acid; (**b**) valeric acid. Data are presented as the mean ± SEM.

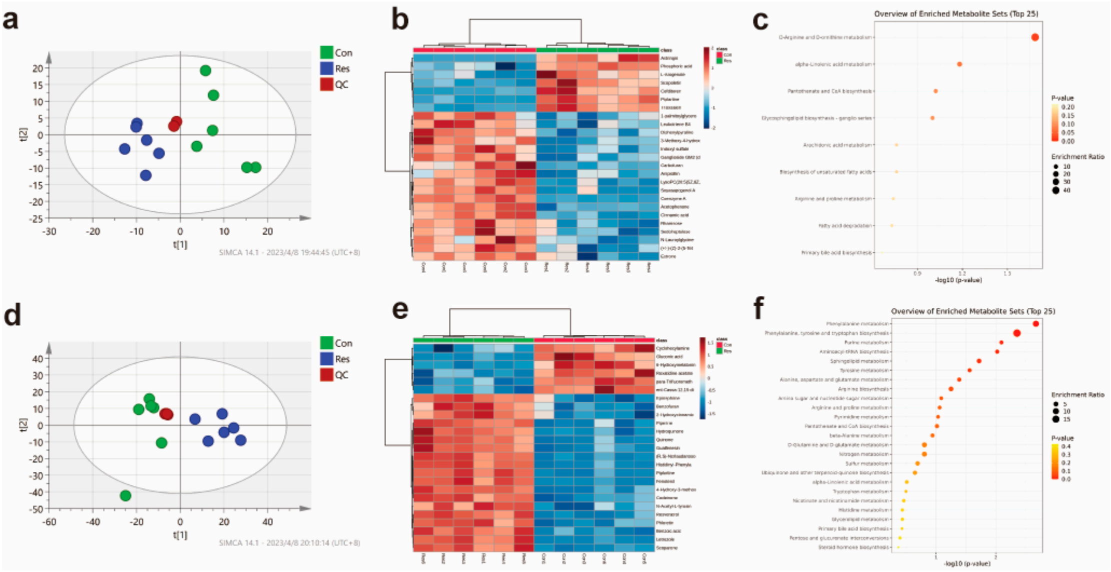

Figure S4. Effects of Res on serum and fecal metabolome at T2 in dogs: (**a**) PCA plot of serum metabolome; (**b**) heatmaps of top 25 differential metabolites in serum calculated by Student's *t*-test; (**c**) KEGG pathways enrichment analysis of differential metabolites in serum; (**d**) PCA plot of fecal metabolome; (**e**) heatmap of top 25 differential metabolites in feces calculated by Student's *t*-test; (**f**) KEGG pathways enrichment analysis of differential metabolites in feces.

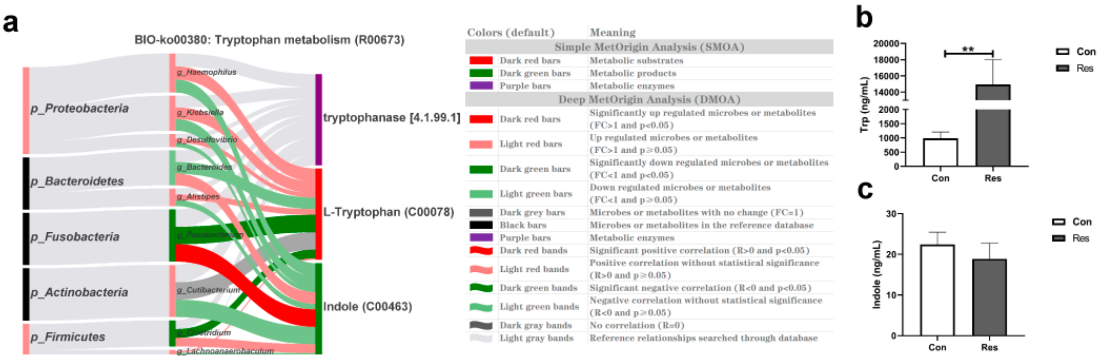

Figure S5. Res regulated tryptophan metabolism by impacting specific microbes in the gut at T2 in the dogs: (**a**) Sankey network of the differential fecal microbiota and tryptophan metabolic pathway; (**b**) concentrations of Trp in the feces; (**c**) concentrations of indole in the feces. Data are presented as the mean ± SEM. The symbol (*) indicates a statistically significant difference between two groups (** $P < 0.01$).

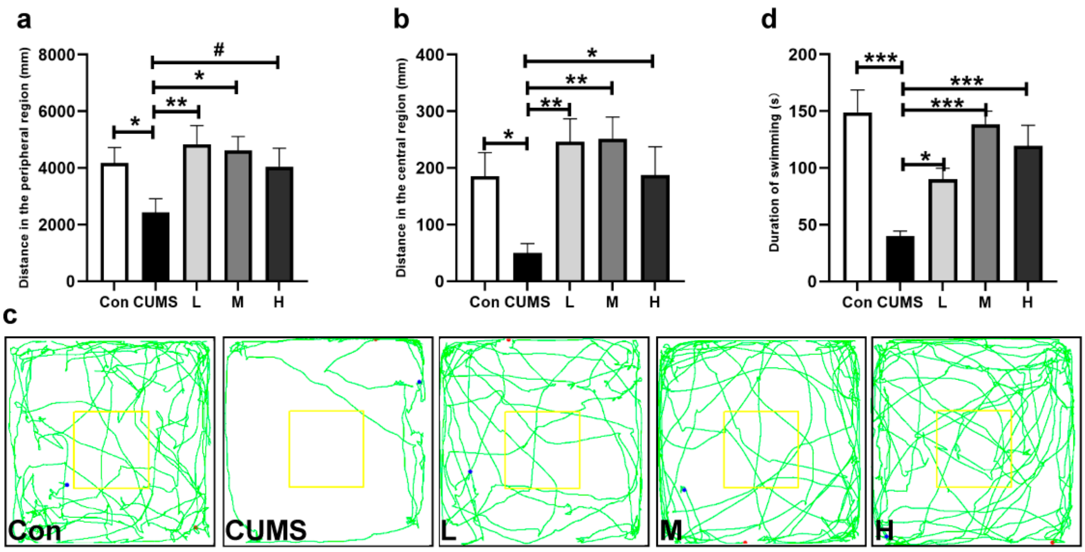

Figure S6. Effects of Res on the OFT and FST in the mice: (**a**) distance in the peripheral region in the OFT; (**b**) distance in the central region in the OFT; (**c**) trajectory of mice in the OFT; (**d**) duration of swimming in the FST. Data are presented as the mean ± SEM. The symbol (*) indicates a statistically significant difference between two groups (*$P < 0.05$, ** $P < 0.01$, and *** $P < 0.001$), and the symbol (#) represents a difference tendency ($P < 0.10$).

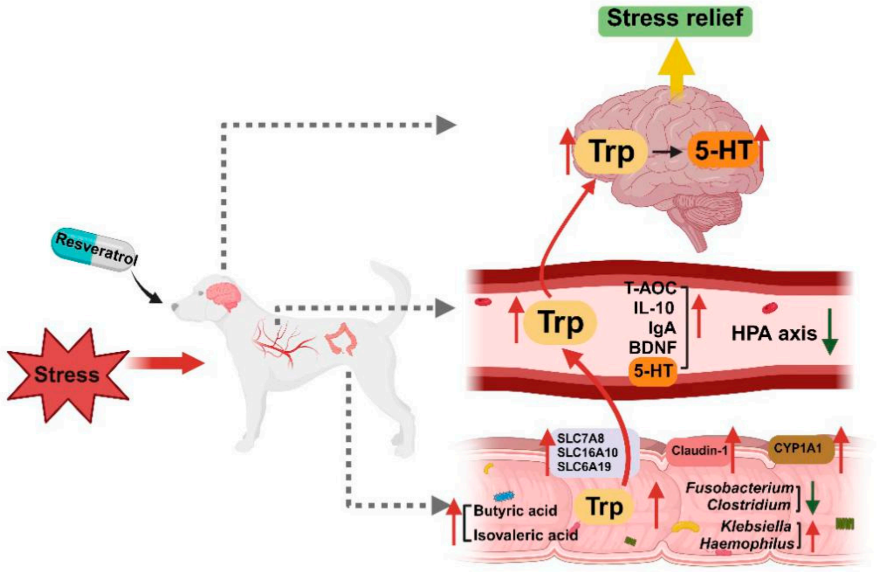

Figure S7. Resveratrol ameliorated chronic stress in kennel dogs and mice by regulating gut microbiome and metabolome related to tryptophan metabolism. (**a**) Trp, tryptophan; (**b**) 5-HT, 5-hydroxytryptamine; (**c**) T-AOC, total antioxidative capacity; (**d**) IL-10, interleukin 10; (**e**) IgA, immunoglobulin A; (**f**) BDNF, brain-derived neurotrophic factor; (**g**) HPA, hypothalamic–pituitary–adrenal.
